# Cost-Effectiveness Analysis of Smoking Cessation Interventions in the United Kingdom Accounting for Major Neuropsychiatric Adverse Events

**DOI:** 10.1016/j.jval.2020.12.012

**Published:** 2021-06

**Authors:** Edna Keeney, Nicky J. Welton, Matt Stevenson, Michael N. Dalili, José A. López-López, Deborah M. Caldwell, David M. Phillippo, Marcus R. Munafò, Kyla H. Thomas

**Affiliations:** 1 Population Health Sciences, Bristol Medical School, University of Bristol, Bristol, England, UK; 2 Health Economics and Decision Science, School of Health and Related Research, University of Sheffield, Sheffield, England, UK; 3 School of Psychological Science, University of Bristol, Bristol, England, UK; 4 MRC Integrative Epidemiology Unit at the University of Bristol, Bristol, England, UK; 5 Department of Basic Psychology & Methodology, Faculty of Psychology, University of Murcia, Murcia, Spain

**Keywords:** cost-effectiveness, economic model, smoking cessation, value of information

## Abstract

**Objectives:**

Smoking is a leading cause of death worldwide. Cessation aids include varenicline, bupropion, nicotine replacement therapy (NRT), and e-cigarettes at various doses (low, standard and high) and used alone or in combination with each other. Previous cost-effectiveness analyses have not fully accounted for adverse effects nor compared all cessation aids. The objective was to determine the relative cost-effectiveness of cessation aids in the United Kingdom.

**Methods:**

An established Markov cohort model was adapted to incorporate health outcomes and costs due to depression and self-harm associated with cessation aids, alongside other health events. Relative efficacy in terms of abstinence and major adverse neuropsychiatric events was informed by a systematic review and network meta-analysis. Base case results are reported for UK-licensed interventions only. Two sensitivity analyses are reported, one including unlicensed interventions and another comparing all cessation aids but removing the impact of depression and self-harm. The sensitivity of conclusions to model inputs was assessed by calculating the expected value of partial perfect information.

**Results:**

When limited to UK-licensed interventions, varenicline standard-dose and NRT standard-dose were most cost-effective. Including unlicensed interventions, e-cigarette low-dose appeared most cost-effective followed by varenicline standard-dose + bupropion standard-dose combined. When the impact of depression and self-harm was excluded, varenicline standard-dose + NRT standard-dose was most cost-effective, followed by varenicline low-dose + NRT standard-dose.

**Conclusion:**

Although found to be most cost-effective, combined therapy is currently unlicensed in the United Kingdom and the safety of e-cigarettes remains uncertain. The value-of-information analysis suggested researchers should continue to investigate the long-term effectiveness and safety outcomes of e-cigarettes in studies with active comparators.

## Introduction

Cigarette smoking is one of the leading causes of early death in the United Kingdom and worldwide.^1^^,^^2^ Although smoking is now down to fewer than 1 in 6 adults (14%) in the United Kingdom, this still equates to approximately 7.35 million people in the population.^3^ In 2017, 16% of all deaths were attributed to smoking, and 33% of deaths for conditions that can be caused by smoking.^3^ The cost of smoking to the UK National Health Service (NHS) has been estimated at between approximately £2.6 and £5 billion a year,^4^^,^^5^ with the total cost to society in England estimated at approximately £12.9 billion a year.^6^

National Institute for Health and Care Excellence (NICE) public health guidance recommends the use of three medicines: varenicline, bupropion, and nicotine replacement therapy (NRT), as aids to quitting smoking in the United Kingdom.^7^ These medicines can be used at different doses and alone or in combination. Although combinations are used, they are not licensed. E-cigarettes are also currently not licensed in the United Kingdom, although there were an estimated 3.2 million adult users in Great Britain in 2018.^8^

Concerns have been raised about the safety of some smoking cessation medicines, particularly the neuropsychiatric safety of varenicline and buproprion. Severe safety warnings regarding a potential increased risk of neuropsychiatric adverse events (depression, suicidal ideation, and suicidal behavior) in patients prescribed varenicline have previously been issued by regulatory agencies.^9^^,^^10^ These safety warnings were removed in 2016^11^^,^^12^ following the results of a large US trial (EAGLES),^13^ which did not show a significant increase in neuropsychiatric adverse events attributable to varenicline or bupropion relative to nicotine patch or placebo. The language describing serious mental health side effects seen in patients quitting smoking was also removed from the bupropion label.^12^ However concerns have since been raised that the study was under-powered to detect a rare adverse effect such as suicide.^14^ It is therefore important to include the consequences of neuropsychiatric safety on costs and quality of life in an economic evaluation of medicines for smoking cessation.

We use efficacy and safety results from a recent network meta-analysis (NMA) to provide an updated cost-effectiveness analysis of smoking cessation medicines in a UK setting. Our results help to inform the overall risk-benefit evaluation of the different medicines and determine which intervention, or combination of interventions, represents the best “value for money” to the NHS. Unlike previous studies, we compare a range of licensed and unlicensed interventions, and the impact of neuropsychiatric adverse events is incorporated. This will allow patients, prescribers, and regulators to make more informed decisions about intervention choice.

## Methods

The population considered in the decision were smokers in the United Kingdom aged 18 years or over. The interventions compared in the base case were those currently licensed in the United Kingdom: NRT at low, standard, and high dose; bupropion at low and standard dose and varenicline at low and standard dose. As e-cigarettes and combination interventions are not currently licensed as smoking cessation interventions in the United Kingdom, we exclude these in the base-case but include them in a sensitivity analysis. E-cigarettes at low and high dose and the following combinations of interventions were considered in the sensitivity analysis, alongside the licensed interventions: bupropion standard dose + NRT high dose; varenicline low dose + NRT standard dose; varenicline standard dose + NRT standard dose; varenicline standard dose + NRT high dose; and varenicline standard + bupropion standard dose.

Standard practice in the NHS is to offer NRT to smokers attempting to quit, at a dose based on level of cigarette use, including combinations of NRT modes of delivery (for example patch and gum). We therefore use NRT standard dose as the reference intervention for comparison in the cost-effectiveness analysis. The perspective taken is that of the UK NHS for costs and health effects of the individual for outcomes, in line with NICE guidance.^15^ A lifetime time horizon was taken to predict costs and health effects over a participant’s lifetime.

The model structure is based on the Sheffield model by Leaviss and colleagues^16^ used in a 2014 Health Technology Assessment report on the clinical and cost-effectiveness of cytisine compared with varenicline for smoking cessation. This in turn was based on the Benefits of Smoking Cessation on Outcomes (BENESCO) model,^17^ a widely used cost-effectiveness model, which has previously been applied to model the effects of smoking cessation interventions in the United Kingdom, the United States, Germany, France, Belgium, the Netherlands, Finland, Sweden, and South Korea.^18^, ^19^, ^20^, ^21^, ^22^, ^23^, ^24^, ^25^, ^26^, ^27^

The model simulates a cohort of smokers making a quit attempt over time, tracking morbidity and mortality to calculate the costs and benefits associated with different smoking cessation aids. Both the cost of the intervention and costs and health state disutilities associated with the smoking-related illnesses of chronic obstructive pulmonary disease, lung cancer, coronary heart disease, stroke, and asthma are captured. The prevalence, incidence, and mortality from these events are considered to depend on whether a person is a smoker, recent quitter, or long-term quitter, as well as their age and sex. Cohort members accumulate costs and health outcomes each cycle until death. Future costs and benefits are discounted at a rate of 3.5% per annum.^15^

No previous model or variation of the BENESCO model has considered adverse events associated with the interventions themselves. We have incorporated these as a probability of experiencing depression or fatal/nonfatal self-harm in the first year of the intervention. Depression and nonfatal self-harm are represented by a one-off disutility and cost whereas fatal self-harm also results in death. We remove the impact of depression and self-harm in a final sensitivity analysis to test the impact on results.

### Model Inputs

#### Efficacy

The probability of smoking cessation associated with NRT was estimated from Taylor et al (2017).^28^ Taylor et al published a prospective cohort study of electronic medical records from 149 526 patients who were prescribed NRT. At 1 year, 21.2% (695 of 149 526) of those prescribed NRT quit. The probabilities of cessation at one year for the other interventions were then obtained by applying the relative effects estimated in a NMA on sustained abstinence^29^ to the cessation probabilities for NRT. The mean probabilities of 1-year sustained abstinence for all interventions used in the model are shown Appendix Table 1 in the Supplementary Material found at https://doi.org/10.1016/j.jval.2020.12.012 and suggest that varenicline low + NRT standard and varenicline standard + NRT standard have the highest probability of sustained abstinence (44%, 95% Credible Interval (CrI) 0.17-0.74 and 44%, 95% CrI 0.23-0.67, at 1 year, respectively) followed by e-cigarettes at low dose (32%, 95% CrI 0.12-0.63).

The probabilities of depression and self-harm at 1 year associated with NRT standard were estimated from Kotz et al.^30^ This was a retrospective cohort study of 106 759 patients who were prescribed NRT. Of these, 8274 reported having depression giving a probability of 7% and 540 reported self-harm giving a probability of 0.5%. The probabilities of depression and self-harm associated with the other interventions were generated applying the relative effects estimated in a NMA on major adverse neuropsychiatric events (MANE)^29^ to the probabilities of depression and self-harm on NRT. The mean probabilities of depression and self-harm for all interventions used in the model are also shown in Appendix Table 1 in the Supplementary Material found at https://doi.org/10.1016/j.jval.2020.12.012.

Because no data were available in the NMA on MANE (depression and self-harm) for some interventions, assumptions had to made about their relative level of harm. We assumed that NRT low and e-cigarette low have the same level of harm as NRT standard. Similarly, e-cigarette high was assumed to have the same level of harm as NRT high. In addition, bupropion low was assumed to have the same level of harm as bupropion standard and varenicline low + NRT standard the same level of harm as varenicline standard + NRT standard. The assumption that NRT and e-cigarettes have the same impact on psychological outcomes was believed to be reasonable as the active ingredient is the same in both (nicotine). Although a higher dose of bupropion or varenicline may increase the probability of depression or self-harm, no evidence was available to inform this.

The relative effects of abstinence, depression and self-harm were estimated using Bayesian NMA, computed using Markov chain Monte Carlo simulation in OpenBUGS.^31^ Simulated samples for the model were drawn from 60 000 Markov chain Monte Carlo samples from the posterior distributions (following 50 000 burn-in samples after which convergence was deemed satisfactory).^29^

### Prevalence, Incidence, and Mortality Associated With Smoking-Related Diseases

The distribution of the cohort across sex and age categories at the start of the model was designed to reflect the distribution of smokers in the United Kingdom. The proportion of male and female adults and mortality risk in each of the 3 age categories (18-34, 35-64, and 65+ years old) was determined from general population data.^32^^,^^33^ Smoking prevalence data^34^ were applied to these data to calculate the distribution across age and sex groups for a representative sample of 10,000 UK smokers (Appendix Table 2 in the Supplementary Material found at https://doi.org/10.1016/j.jval.2020.12.012).

The prevalence, incidence and mortality from smoking-related diseases in the smoking cohort was estimated based on various literature sources on general UK population figures and risk ratios of these diseases in smokers, recent quitters, and long-run quitters (Appendix Tables 2 and 3 in the Supplementary Material found at https://doi.org/10.1016/j.jval.2020.12.012). Relative risks for the prevalence of each disease in smokers relative to never-smokers were taken from the Statistics on Smoking England 2017 report^35^ for chronic obstructive pulmonary disease, lung cancer, coronary heart disease, and stroke, and Cassino et al^36^ for asthma (Appendix Table 4 in the Supplementary Material found at https://doi.org/10.1016/j.jval.2020.12.012).

### Relapse Rates

Hawkins et al^37^ used British Household Panel Survey data to look at smokers who quit but later relapsed. These data were used to calculate the annual relapse probability for short-run quitters (people for whom it had been less than 5 years since they quit; 0.13, 95% CrI 0.12-0.14) and long-run quitters (people who had quit smoking for >5 years but <10 years; 0.03, 95% CrI 0.02-0.05). The annual relapse probability ≥10 years post cessation (0.0009, 95% CrI 0.0004-0.0015) was based on a study by Krall and colleagues which followed 483 men for up to 35 years.^38^

### Costs and Utilities

Costs and utilities are accumulated in the model by following a cohort of quitters moving between different health states. Estimates for the costs associated with health states came from a range of data sources including a recent report by the Irish Health Information Quality Authority,^39^ the British Heart Foundation’s CVD statistics^40^ and recent UK articles^41^^,^^42^ (Appendix Table 5 in the Supplementary Material found at https://doi.org/10.1016/j.jval.2020.12.012). Uncertainty around cost estimates was incorporated into the probabilistic analysis. These data were assumed to follow a gamma distribution.^43^ A one-off cost was also associated with the initial smoking cessation intervention received. Costs for all interventions including combinations of interventions were based on estimates from the British National Formulary and are shown in Appendix Table 5 in the Supplementary Material found at https://doi.org/10.1016/j.jval.2020.12.012. All costs were inflated to 2019 prices using HM Revenues Monthly Exchange rates for February 2019.^44^

Baseline utility for smokers with no current comorbidity was taken from the general population utility profile estimated by Ara and Brazier using 2003 and 2006 Health Survey for England data.^45^ Disease-specific utility values for smoking-related diseases were estimated from the literature.^42^^,^^45^, ^46^, ^47^, ^48^, ^49^, ^50^, ^51^ Choice of estimates was based on sample size and relevance to a contemporary UK population. The estimates are shown in Appendix Table 5 in the Supplementary Material found at https://doi.org/10.1016/j.jval.2020.12.012.

### How Results Are Reported

We conduct a probabilistic analysis where uncertainty in the model inputs is captured by simulating 5000 times from the assumed distributions of the parameters described in the previous section, using Monte Carlo simulation performed in Excel version 1908 (Microsoft Corporation, Redmond, WA). We report mean lifetime costs and quality adjusted life years (QALYs) for each intervention. Incremental cost-effectiveness ratios (ICERs), which are the ratio of the incremental costs and incremental QALYs, are reported for all interventions compared with NRT low. An intervention is considered to be dominated if it provides less mean QALYs at a higher mean cost than another intervention, and extendedly dominated if it provides less mean QALYs at a higher mean cost than a weighted average of 2 alternative interventions.

We also report the expected net benefit with 95% confidence interval for all interventions, where expected net benefit = mean QALYs × WTP – mean costs, where WTP is the willingness-to-pay threshold. A WTP threshold of £20 000 is chosen as this is the lower threshold referred to in the NICE reference case.^52^

Cost-effectiveness acceptability curves (CEACs) are plotted alongside rank-o-grams. CEACs plot the probability that each intervention is the most cost-effective (highest NB). Rank-o-grams show the distribution of the probabilities that each intervention is most cost-effective, second most cost-effective, third most cost-effective, and so on for each of the 14 interventions, at a fixed willingness to pay threshold, in this case £20 000 per QALY.^53^ The x-axis reports each of the possible ranks, for which position 1 means that the intervention is most cost-effective. The y-axis shows the probability that each intervention has been ranked at each of the possible positions and therefore fully encapsulates the uncertainty in the intervention rankings. We also report the median rank and interquartile range for each intervention.

We explore how uncertainty in the model inputs impacts on the intervention considered to be optimal using value of information (VoI) methods.^54^ The expected value of perfect information (EVPI) and expected value of partially perfect information (EVPPI) give an upper bound on the benefit in reducing uncertainty in all or a subset of the model inputs, respectively. EVPPI can be used to identify which parameters the decision is most sensitive to. EVPI and EVPPI are computed per person for a willingness-to-pay per QALY threshold of £20 000 and multiplied by the estimated number of smokers attempting to quit in England of 274 021^3^ to obtain population-level EVPI and EVPPI. The Sheffield Accelerated Value of Information web application^55^ was used to compute EVPPI for subsets of parameters.^56^

Finally, we present 2 sensitivity analyses. One expanding the analysis to include e-cigarettes and combinations of interventions and another where the impacts of depression and self-harm in the model are removed, so the results are driven by abstinence from smoking alone.

## Results

Table 1 shows the primary results of the base case analysis: per smoker expected total discounted costs and QALYs for all UK-licensed interventions during over the simulation sample in a probabilistic analysis. Interventions are ordered by increasing expected total cost with NRT low having the lowest expected total cost, and varenicline low having the highest expected total cost. Varenicline standard has the highest expected QALYs followed by NRT standard. NRT low has the lowest expected QALYs.

**Table 1:** Expected total costs, expected total utilities, ICERs, and expected net benefit at a £20 000 willingness-to-pay threshold based on UK licensed interventions only.

Intervention	Total costs	Total QALYs	ICER	ENB (95% CI) (£)	Median rank (IQR)
NRT low	£10 259	10.934		0	7 (6-7)
Bupropion low	£10 283	11.038	Extendedly dominated	2056 (2010, 2102)	5 (4-6)
NRT STD	£10 292	11.119	£32	3663 (3617, 3710)	2 (1-3)
Bupropion STD	£10 304	11.033	Dominated	1937 (1902, 1971)	5 (4-5)
NRT high	£10 309	11.092	Dominated	3092 (3053, 3131)	3 (2-4)
Varenicline STD	£10 413	11.127	£15 665	3697 (3659, 3734)	2 (1-3)
Varenicline low	£10 440	10.959	Dominated	308 (211, 405)	6 (4-7)

CI indicates confidence interval; ENB*,* expected net benefit; ICER, Incremental cost-effectiveness ratios; IQR, interquartile range; NRT, nicotine replacement therapy; QALY, quality adjusted life years; STD, standard.

All interventions are dominated by varenicline standard apart from NRT low and NRT standard. Bupropion low is extendedly dominated by NRT standard as its ICER is higher than that of NRT standard, the next most effective alternative. If the decision maker is only willing to pay up to £32 per QALY, NRT low would be considered the most cost-effective intervention. At any willingness to pay value between £32 and £15 665, NRT standard is most cost-effective and at a willingness to pay value above £15 665, varenicline standard is most cost effective. Varenicline standard has the highest expected net benefit (£3697), followed by NRT standard (£3663).

**Figure 1:**
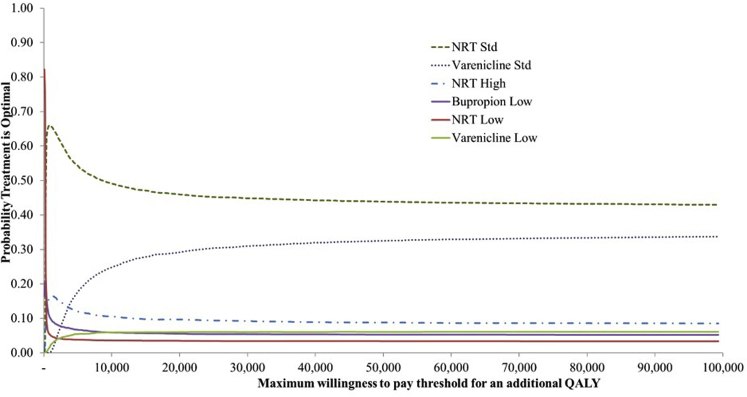
CEAC. Probability treatment is optimal plotted against different willingness-to-pay per unit increase in utility (ceiling ratio). Based on 5000 Monte Carlo simulations.

We present the uncertainty surrounding the cost-effectiveness of the various interventions, using a CEAC. Figure 1 shows that at any willingness-to-pay value above £100, NRT standard has the highest probability of being cost-effective, followed by varenicline standard. At any threshold above £20 000 the probability of NRT standard being the most cost-effective intervention is never more than 50%, indicating a degree of uncertainty in the optimal intervention.

**Figure 2:**
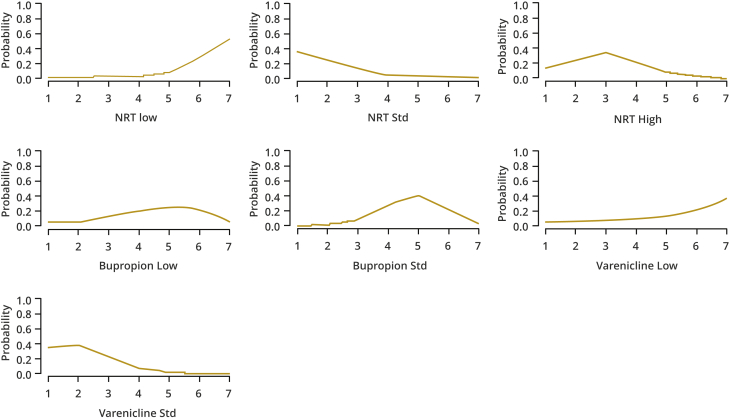
Rank-o-grams showing the probability that each intervention is ranked 1st, 2nd, … etc. based on Net Benefit at a willingness to pay threshold of £20,000 per QALY.

The rank-o-grams presented in Figure 2 and median ranks in Table 1 show that both NRT standard and varenicline standard have a high probability of being among the most cost-effective interventions. Conversely, varenicline low and NRT low show higher probabilities of being among the lowest ranking interventions.

### Value-of-Information Analysis

Table 2 shows the results of the VoI analyses for the base-case model at a willingness to-pay per QALY threshold of £20 000. The per-quitter EVPI is £708 and the population EVPI, representing all of the smokers attempting to quit in England, is £194 million for a 1-year time horizon and £971 million for a 5-year time horizon. These values are substantial and suggest that future research studies to reduce parameter uncertainty in the model would be valuable as the decision is clearly sensitive to uncertainty in the model inputs. There is a high value per smoker in reducing uncertainty in all of the abstinence probabilities (£473) but more value in reducing uncertainty in all of the adverse events probabilities (£575). EVPPI is lower for cost parameters (£58) than for utility parameters (£118).

**Table 2:** Expected value of perfect information and EVPPI for various subsets of model parameters, at a £20,000 willingness-to-pay value per QALY.

Model parameter subsets	EVPPI per smoker attempting to quit (£)	1-y population EVPPI (£ million)	5-y population EVPPI (£ million)
All (EVPI)	709	194	971
All costs	58	16	79
All utilities	118	32	161
All costs and utilities	403	110	552
All abstinence probabilities	473	130	648
All depression and self-harm probabilities	575	157	787
NRT STD vs varenicline STD (probabilities, costs and utilities)	544	149	745
NRT STD vs varenicline STD (probabilities only)	528	145	723

EVPPI indicates expected value of partially perfect information; NRT, nicotine replacement therapy; QALY, quality adjusted life years; STD, standard.

We explored the EVPPI of a new trial comparing the 2 interventions with the highest expected net benefit, NRT standard and varenicline standard, which would provide information on the effectiveness of the interventions, costs, and utilities. This gives a per-quitter EVPPI of £544 and a population EVPPI of £149 million for a 1-year time horizon and £746 million for a 5-year time horizon. Restricting to the collection of intervention effects only reduces this value marginally to £528 per quitter, suggesting that it is most important to collect information on probabilities of abstinence and adverse events.

### Sensitivity Analyses

Table 3 shows the primary results of the sensitivity analysis where all licensed and unlicensed interventions are compared. In this case all interventions apart from NRT low are dominated by e-cigarette low, which is more effective, in terms of increased QALYs, and less expensive than the other interventions. At a willingness-to-pay threshold of £20 000, e-cigarette low has the highest expected net benefit (£7085), followed by varenicline standard + bupropion standard (£6756), and varenicline standard + NRT standard (£6591).

**Table 3:** Expected total costs, expected total utilities, ICERs and expected net benefit at a £20 000 willingness-to-pay threshold.

Intervention	Total costs	Total QALYs	ICER	ENB (95% CI) (£)	Median rank (IQR)
NRT low	£10 259	10.934		0	13 (12-14)
E-cigarette low	£10 279	11.290	£56	7085 (6964, 7205)	3 (1-6)
Bupropion low	£10 283	11.038	Dominated	2056 (2010, 2102)	11 (9-12)
NRT STD	£10 292	11.119	Dominated	3663 (3617, 3710)	6 (5-8)
Bupropion std	£10 304	11.033	Dominated	1937 (1902, 1971)	11 (10-12)
NRT high	£10 309	11.092	Dominated	3092 (3053, 3131)	8 (7-10)
E-cigarette high	£10 319	11.189	Dominated	5036 (4967, 5104)	5 (3-7)
Bupropion std + NRT high	£10 346	11.128	Dominated	3786 (3710, 3862)	7 (4-11)
Varenicline STD	£10 413	11.127	Dominated	3697 (3659, 3734)	7 (6-8)
Varenicline STD + bupropion STD	£10 437	11.281	Dominated	6756 (6669, 6843)	3 (2-5)
Varenicline low	£10 440	10.959	Dominated	308 (211, 405)	12 (10-14)
Varenicline STD + NRT high	£10 467	11.117	Dominated	3440 (3370, 3509)	8 (5-11)
Varenicline low + NRT STD	£10 587	11.273	Dominated	6454 (6313, 6595)	3 (2-8)
Varenicline STD + NRT STD	£10 587	11.280	Dominated	6591 (6472, 6710)	3 (2-6)

CI indicates confidence interval; ENB*,* expected net benefit; EVPPI indicates expected value of partially perfect information; ICER, incremental cost-effectiveness ratio; IQR, interquartile range; NRT, nicotine replacement therapy; QALY, quality adjusted life years; STD, standard.

The median ranks and rank-o-grams presented in Appendix Table 3 and Figure 1 of the Supplementary Material found at https://doi.org/10.1016/j.jval.2020.12.012 demonstrate the uncertainty in these results. In the rank-o-grams the lines are relatively flat for most interventions showing that there is no strong probability that any will be most or least cost-effective at a £20 000 per QALY threshold. The exception is NRT low which shows a high probability of being among the least cost-effective interventions. There is a similar trend for bupropion low, bupropion standard, and varenicline low. The reverse trend is seen for e-cigarette low, varenicline low + NRT standard, varenicline standard + NRT standard and varenicline standard + bupropion standard, which all have the highest median rank of 3.

The VoI results based on this analysis are shown Appendix Table 8 of the Supplementary Material found at https://doi.org/10.1016/j.jval.2020.12.012. We again explored the EVPPI of a new trial comparing the two interventions with the highest expected net benefit, e-cigarette low and varenicline standard + bupropion standard. This gives a per-quitter EVPPI of £2342 and a population EVPPI of £642 million for a 1-year time horizon and £3209 million for a 5-year time horizon. Restricting to the collection of intervention effects only reduces this value marginally to £1676 per quitter, suggesting that a trial comparing e-cigarettes low to an active comparator such as varenicline standard + bupropion standard or NRT standard is likely to be a cost-effective investment.

Table 4 shows the primary results of the sensitivity analysis where all interventions are compared but the impact of depression and self-harm is removed from the model. In this case, e-cigarette low is replaced by varenicline standard + NRT standard as the intervention with the highest expected net benefit (£9895), followed by varenicline low + NRT standard (£9759).

**Table 4:** Expected total costs, expected total utilities, ICERs and expected net benefit at a £20 000 willingness-to-pay threshold based on abstinence alone.

Intervention	Total costs	Total QALYs	ICER	ENB	Median rank (IQR)
Bupropion low	£10 219	11.135		3159 (3114, 3204)	11 (9-12)
NRT low	£10 231	10.977	Dominated	0	14 (14-14)
NRT high	£10 238	11.198	Extendedly dominated	4400 (4365, 4434)	7 (6-8)
Bupropion STD	£10 240	11.130	Dominated	3041 (3008, 3073)	11 (10-12)
E-cigarette high	£10 248	11.295	Extendedly dominated	6335 (6269, 6401)	4 (3-6)
E-cigarette low	£10 250	11.332	£159	7072 (6958, 7187)	4 (2-8)
NRT STD	£10 264	11.162	Dominated	3657 (3628, 3686)	9 (8-10)
Bupropion std + NRT high	£10 319	11.168	Dominated	3721 (3647, 3794)	10 (6-13)
Varenicline low	£10 320	11.138	Dominated	3120 (3076, 3164)	11 (9-12)
Varenicline STD	£10 327	11.254	Dominated	5434 (5399, 5469)	5 (4-6)
Varenicline STD + NRT high	£10 402	11.214	Dominated	4556 (4492, 4619)	10 (6-13)
Varenicline STD + bupropion STD	£10 415	11.314	Dominated	6558 (6475, 6642)	4 (3-7)
Varenicline low + NRT STD	£10 446	11.476	Extendedly dominated	9759 (9636, 9882)	2 (1-4)
Varenicline STD + NRT STD	£10 447	11.483	£1,302	9895 (9799, 9991)	2 (1-3)

ENB indicates expected net benefit; ICER, Incremental cost-effectiveness ratios; IQR, interquartile range; NRT, nicotine replacement therapy; QALY, quality adjusted life years; STD, standard.

## Discussion

Our results show that, in the base case, when the analysis is limited to UK-licensed interventions (and neuropsychiatric events included), varenicline standard is most cost-effective at any willingness to pay value above £15 665. When all interventions are included, e-cigarette low is most cost-effective at any willingness to pay value above £56. At a willingness to pay of £20 000, e-cigarette low, varenicline standard + bupropion standard, and varenicline standard + NRT standard were found to be most cost-effective. The results have also shown that including the safety outcomes of depression and self-harm makes a difference. When these are not accounted for, varenicline standard + NRT standard is most cost-effective at any willingness-to-pay value above £1302. VoI analyses have indicated that a trial comparing e-cigarettes to an active comparator such as varenicline standard + bupropion standard or NRT standard is likely to be a cost-effective investment. Although more research is needed, these results suggest that decision and policymakers should consider licensing of combination interventions and e-cigarettes. The prescription of these interventions on the NHS could lead to better outcomes at reduced costs.

No previous CEA was identified that compared a similar range of interventions, including standard licensed interventions, combination therapies, and e-cigarettes, or incorporating safety outcomes. A recent systematic review of CEAs^39^ identified 4 studies published in the past 10 years which compared varenicline, bupropion, or NRT to each other or to standard of care.^20^^,^^26^^,^^57^^,^^58^ All but one of these studies^58^ also used the BENESCO model but none adjusted to account for safety outcomes. In agreement with our findings, the studies consistently found varenicline to be the most cost-effective intervention. Our model differs from the previous models by including adverse events for depression and self-harm. We explored the impact of excluding these adverse events in a sensitivity analysis. We found that when considering both licensed and unlicensed interventions the combination of varenicline and NRT was most cost-effective when adverse events were not included, but that e-cigarettes low and the combination of varenicline standard and bupropion standard were more cost-effective when adverse events were included.

One previous study was identified which compared the cost-effectiveness of e-cigarettes to NRT in stop smoking services in England.^59^ Similar to our study where an ICER of £56 was calculated for e-cigarette low compared with NRT low, this study found an ICER of £65 per QALY gained by using e-cigarettes in comparison with NRT. However, to our knowledge, our study is the first to assess the cost-effectiveness of e-cigarettes compared with all other interventions in the United Kingdom.

There were several data limitations including a lack of comparative evidence on subsequent quit attempts in these interventions. The model assumes that, after a failed first attempt, smokers remain so until death, when in reality people often make several quit attempts before they are successful. We would expect our findings to be robust to this however as long as the likelihood of a successful subsequent quit attempt does not depend on the initial intervention.

Another data limitation is the assumption that the risk ratios of developing or dying from smoking-related diseases in current smokers and former smokers compared to nonsmokers are equal to the risk ratios of having smoking related-diseases. We considered this reasonable given that no alternative sources of information on relative incidence or mortality within the specified age and sex categories could be identified. Longitudinal studies measuring these outcomes for the different smoker categories would be useful to inform future models.

This distribution of the cohort across sex and age categories at the start of the model was designed to reflect the distribution of smokers in the United Kingdom. One issue is that this is not necessarily the same as the distribution of smokers making a quit attempt. Another is that data availability meant that this cohort needed to be grouped into broad age categories (18-34, 35-64, >65 years old) which have been assigned the same prevalence, incidence, and probability of mortality from diseases within each category. It is likely, therefore, that greater variation exists within these categories than is being accounted for. A study measuring patient characteristics of those seeking intervention to make a quit attempt would also be useful to update the model to better reflect the population of interest.

With no medically licensed e-cigarettes available in the United Kingdom it is difficult to estimate a prescribing cost if they were to be prescribed on the NHS. The best evidence we could find on this was from the Irish Health Information Quality Authority HTA,^60^ which estimated a 12-week supply of e-cigarettes as €93.80. Current high-street/internet prices may be considerably lower than this; however, it is unclear whether the NHS would be able to access these lower prices. If a lower price could be accessed this could only have the impact of increasing the cost-effectiveness of e-cigarettes compared to the other interventions.

Despite the large number of studies included in the NMAs (161 in the NMA on sustained abstinence and 73 in the NMA on MANEs), comparisons between active interventions were almost exclusively informed by indirect evidence. This resulted in imprecisely estimated effects and wide confidence intervals in some cases. In our model we included depression and self-harm as adverse events. The label for varenicline however mentions depressed mood. We did not find sufficient data to include this in the model, and so instead included depression, which was reported in the RCTs and cohort studies identified in our searches. In addition, as no data were available, assumptions had to made about the relative effectiveness of several interventions for the outcomes of depression and self-harm. More studies comparing the impact of different doses and combinations of these interventions on abstinence and psychological outcomes would be useful to inform the model. In particular, more trials comparing e-cigarettes with active interventions are needed to assess their short- and long-term safety.

### Conclusions

This study used up to date information to give an estimate of the most cost-effective intervention for smoking cessation in the United Kingdom. This analysis has shown that in the base case, among licensed interventions, varenicline standard or NRT standard appear to be most cost-effective. When all licensed and unlicensed comparators are included, e-cigarette low, varenicline standard + bupropion standard, or varenicline standard + NRT standard appear to be most cost-effective. When the impact of the safety outcomes of depression and self-harm is excluded, varenicline standard + NRT standard and varenicline low + NRT standard are the most cost-effective interventions.

We recommend that researchers continue to investigate the use of e-cigarettes for smoking cessation, particularly with respect to long-term effectiveness and safety outcomes, preferably in studies with active interventions as comparators. Our VoI analysis suggested that a large adequately powered and well-conducted trial comparing E-cigarettes to an active comparator such as varenicline standard + bupropion standard or NRT standard is likely to be a cost-effective use of resources.

## Supplemental Material

Appendix Figure E1 and Tables E1-E8
