# Supplementary material for: Cost-Effectiveness Analysis of Smoking Cessation Interventions in the United Kingdom Accounting for Major Neuropsychiatric Adverse Events
Source: Value Health. 2021 Jun;24(6):780–8. doi: 10.1016/j.jval.2020.12.012 (PMC8177405; doi:10.1016/j.jval.2020.12.012)

*Table 1. Absolute probabilities of abstinence, depression and self-harm*

|  | **Abstinence** | **95% CrI** | **Depression** | **95% CrI** | **Self-harm** | **95% CrI** |
| --- | --- | --- | --- | --- | --- | --- |
| Varenicline Low + NRT Std | 0.44 | (0.17-0.74) | 0.36 | (0.09-0.77) | 0.042 | (0.006-0.158) |
| Varenicline Std + NRT Std | 0.44 | (0.23-0.67) | 0.36 | (0.09-0.77) | 0.042 | (0.006-0.158) |
| E-Cigarette Low | 0.32 | (0.12-0.63) | 0.07 | (0-0.39) | 0.006 | (0-0.036) |
| Varenicline Std & Bupropion Std | 0.31 | (0.15-0.52) | 0.06 | (0–0.24) | 0.004 | (0-0.018) |
| E-Cigarette High | 0.31 | (0.18-0.46) | 0.19 | (0.1-0.3) | 0.013 | (0.006-0.024) |
| Varenicline Std | 0.27 | (0.23-0.32) | 0.22 | (0.16-0.31) | 0.016 | (0.011-0.025) |
| Varenicline Std + NRT High | 0.24 | (0.13-0.4) | 0.17 | (0.06-0.37) | 0.013 | (0.003-0.033) |
| NRT High | 0.24 | (0.19-0.28) | 0.19 | (0.1-0.3) | 0.013 | (0.006-0.024) |
| Bupropion Std + NRT High | 0.22 | (0.09-0.43) | 0.08 | (0.01-0.22) | 0.005 | (0.001-0.016) |
| NRT Std | 0.21 | (0.21-0.21) | 0.07 | (0-0.39) | 0.006 | (0-0.036) |
| Varenicline Low | 0.20 | (0.12-0.29) | 0.29 | (0.02-0.78) | 0.038 | (0.003-0.217) |
| Bupropion Low | 0.19 | (0.12-0.29) | 0.17 | (0.11-0.24) | 0.012 | (0.007-0.018) |
| Bupropion Std | 0.19 | (0.15-0.23) | 0.17 | (0.11-0.24) | 0.012 | (0.007-0.018) |
| NRT Low | 0.09 | (0.01-0.24) | 0.07 | (0-0.39) | 0.006 | (0-0.036) |

*Table 2. Population, Prevalence, Incidence and Mortality estimates for general population*

| **Model parameter** | **Source** | **M 18-34** | **M 35-64** | **M 65+** | **F 18-34** | **F 35-64** | **F 65+** |
| --- | --- | --- | --- | --- | --- | --- | --- |
| General population (n) | ONS 2016 | 7,459,224 | 12,446,202 | 5,359,995 | 7,288,586 | 12,759,446 | 6,454,090 |
| Smoking prevalence (% of population) | ONS 2016 | 22.48% | 18.75% | 8.80% | 17.85% | 15.06% | 7.80% |
| Annual probability of all-cause mortality | ONS 2016 | 0.07% | 0.40% | 4.40% | 0.03% | 0.26% | 4.13% |
| Prevalence of COPD | British Lung Foundation, 2012(59) | 0.00% | 1.00% | 7.00% | 0.00% | 1.00% | 7.00% |
| Prevalence of Lung Cancer | Maddams et al, 2009(60) | 0.00% | 0.10% | 0.70% | 0.00% | 0.10% | 0.30% |
| Prevalence of CHD | Health Survey for England 2016 (61) | 0.20% | 4.50% | 20.90% | 0.40% | 1.90% | 12.00% |
| Prevalence of Stroke | Bhatnagar 2015(62) | 0.10% | 1.80% | 10.60% | 0.10% | 1.40% | 8.40% |
| Prevalence of Asthma | British Lung Foundation, 2012(62) | 19.00% | 12.00% | 11.00% | 19.00% | 12.00% | 11.00% |
| Incidence of COPD | Pfizer 2007(63) | 0.00% | 0.01% | 0.30% | 0.00% | 0.01% | 0.20% |
| Incidence of Lung Cancer | Office for National Statistics 2005(64) | 0.00% | 0.04% | 0.40% | 0.00% | 0.04% | 0.33% |
| Incidence of CHD (first non-fatal event) | British Heart Foundation 2006. CHD Statistics Fact Sheet(65) | 0.00% | 0.08% | 0.80% | 0.00% | 0.02% | 0.60% |
| Incidence of CHD (any non-fatal event) | Volmink 1998(66) | 0.00% | 0.12% | 1.40% | 0.00% | 0.03% | 0.90% |
| Incidence of Stroke (first non-fatal event) | ONS 2001 Health Statistics Quarterly(67) | 0.00% | 0.15% | 0.65% | 0.00% | 0.10% | 0.60% |
| Incidence of Stroke (any non-fatal event) | ONS 2001 Health Statistics Quarterly | 0.00% | 0.20% | 1.00% | 0.00% | 0.14% | 1.00% |
| Incidence of Asthma | Asthma UK(68) | 0.06% | 0.05% | 0.06% | 0.06% | 0.05% | 0.05% |
| Mortality from COPD | CVD Statistics 2017, BHF(37) | 0.00% | 1.45% | 4.22% | 0.00% | 1.26% | 3.63% |
| Mortality from Lung Cancer | CVD Statistics 2017, BHF | 0.00% | 29.30% | 48.80% | 0.00% | 23.80% | 34.30% |
| Mortality from Stroke (first non-fatal event) | Assumption. The same split between first event and all events is assumed for first/subsequent for mortality as for incidence. | 0.41% | 0.51% | 1.78% | 0.14% | 0.58% | 2.74% |
| Mortality from Stroke (any non-fatal event) | CVD Statistics 2017, BHF. | 0.60% | 0.68% | 2.74% | 0.20% | 0.81% | 4.57% |
| Mortality from CHD (first non-fatal event) | Assumption. The same split between first event and all events as used in a previous manufacturer’s STA submission(63) is assumed for first/subsequent | 0.53% | 0.92% | 1.71% | 0.06% | 0.88% | 2.23% |
| Mortality from CHD (any non-fatal event) | CVD Statistics 2017, BHF. | 0.70% | 1.37% | 2.90% | 0.08% | 0.88% | 3.05% |

*Table 3. Population, Prevalence, Incidence and Mortality estimates for smokers, recent quitters and long-run quitters*

| Model parameter | M18-34 | M35-64 | M65+ | F18-34 | F35-64 | F65+ |
| --- | --- | --- | --- | --- | --- | --- |
| Prevalence of COPD in UK smokers | 0.00% | 2.10% | 12.10% | 0.00% | 2.30% | 15.20% |
| Prevalence of Lung Cancer in UK smokers | 0.00% | 0.30% | 2.50% | 0.00% | 0.20% | 1.30% |
| Prevalence of CHD in UK smokers | 0.20% | 9.10% | 29.00% | 0.40% | 4.60% | 18.60% |
| Prevalence of Stroke in UK smokers | 0.10% | 4.40% | 17.80% | 0.10% | 4.00% | 14.60% |
| Prevalence of Asthma in UK smokers | 24.80% | 12.10% | 12.10% | 25.20% | 12.20% | 12.10% |
| Annual incidence of COPD in smokers | 0.00% | 0.02% | 0.52% | 0.00% | 0.02% | 0.44% |
| Annual incidence of Lung Cancer in smokers | 0.00% | 0.13% | 1.35% | 0.00% | 0.14% | 1.34% |
| Annual incidence of CHD (first non-fatal event) in smokers | 0.00% | 0.16% | 1.11% | 0.00% | 0.05% | 0.93% |
| Annual incidence of Stroke (first non-fatal event) in smokers | 0.00% | 0.36% | 1.09% | 0.00% | 0.28% | 1.04% |
| Annual incidence of CHD (any non-fatal event) in smokers | 0.00% | 0.24% | 1.94% | 0.00% | 0.07% | 1.40% |
| Annual incidence of Asthma in smokers | 0.08% | 0.05% | 0.07% | 0.08% | 0.05% | 0.06% |
| Annual incidence of Stroke (any non-fatal event) in smokers | 0.00% | 0.49% | 1.68% | 0.00% | 0.40% | 1.73% |
| Annual incidence of COPD in recent quitters | 0.00% | 0.02% | 0.47% | 0.00% | 0.02% | 0.43% |
| Annual incidence of Lung Cancer in recent quitters | 0.00% | 0.05% | 0.50% | 0.00% | 0.05% | 0.48% |
| Annual incidence of CHD (first non-fatal event) in recent quitters | 0.00% | 0.09% | 0.83% | 0.00% | 0.02% | 0.64% |
| Annual incidence of Stroke (first non-fatal event) in recent quitters | 0.00% | 0.11% | 0.63% | 0.00% | 0.08% | 0.61% |
| Annual incidence of CHD (any non-fatal event) in recent quitters | 0.00% | 0.13% | 1.46% | 0.00% | 0.03% | 0.96% |
| Annual incidence of Asthma in recent quitters | 0.06% | 0.05% | 0.06% | 0.06% | 0.05% | 0.05% |
| Annual incidence of Stroke (any non-fatal event) in recent quitters | 0.00% | 0.14% | 0.97% | 0.00% | 0.11% | 1.02% |
| Annual incidence of COPD in long-run quitters | 0.00% | 0.00% | 0.03% | 0.00% | 0.00% | 0.04% |
| Annual incidence of Lung Cancer in long-run quitters | 0.00% | 0.05% | 0.50% | 0.00% | 0.05% | 0.48% |
| Annual incidence of CHD (first non-fatal event) in long-run quitters | 0.00% | 0.05% | 0.69% | 0.00% | 0.01% | 0.53% |
| Annual incidence of Stroke (first non-fatal event) in long-run quitters | 0.00% | 0.10% | 0.58% | 0.00% | 0.06% | 0.53% |
| Annual incidence of CHD (any non-fatal event) in long-run quitters | 0.00% | 0.07% | 1.21% | 0.00% | 0.02% | 0.80% |
| Annual incidence of Asthma in long-run quitters | 0.06% | 0.05% | 0.06% | 0.06% | 0.05% | 0.05% |
| Annual incidence of Stroke (any non-fatal event) in long-run quitters | 0.00% | 0.13% | 0.89% | 0.00% | 0.09% | 0.89% |
| Annual mortality from COPD in smokers | 0.00% | 3.05% | 7.28% | 0.00% | 2.88% | 7.89% |
| Annual mortality from Lung Cancer in smokers | 0.00% | 29.30% | 48.80% | 0.00% | 23.80% | 34.30% |
| Annual mortality from CHD (first non-fatal event) in smokers | 0.53% | 1.85% | 2.37% | 0.06% | 2.14% | 3.46% |
| Annual mortality from CHD (any non-fatal event) in smokers | 0.70% | 2.76% | 4.02% | 0.08% | 2.14% | 4.74% |
| Annual mortality form Stroke (first non-fatal event) in smokers | 0.41% | 1.24% | 3.00% | 0.14% | 1.64% | 4.75% |
| Annual mortality from Stroke (any non-fatal event) in smokers | 0.60% | 1.65% | 4.61% | 0.20% | 2.29% | 7.92% |
| Annual mortality from COPD in recent quitters | 0.00% | 2.79% | 6.65% | 0.00% | 2.81% | 7.71% |
| Annual mortality from Lung Cancer in recent quitters | 0.00% | 29.30% | 48.80% | 0.00% | 23.80% | 34.30% |
| Annual mortality from CHD (first non-fatal event) in recent quitters | 0.53% | 0.99% | 1.78% | 0.06% | 0.98% | 2.37% |
| Annual mortality form Stroke (first non-fatal event) in recent quitters | 0.41% | 0.36% | 1.73% | 0.14% | 0.47% | 2.80% |
| Annual mortality from CHD (any non-fatal event) in recent quitters | 0.70% | 0.99% | 2.51% | 0.08% | 0.98% | 3.25% |
| Annual mortality from Stroke (any non-fatal event) in recent quitters | 0.60% | 0.48% | 2.67% | 0.20% | 0.66% | 4.67% |
| Annual mortality from COPD in long-run quitters | 0.00% | 0.20% | 0.40% | 0.00% | 0.20% | 0.70% |
| Annual mortality from Lung Cancer in long-run quitters | 0.00% | 29.30% | 48.80% | 0.00% | 23.80% | 34.30% |
| Annual mortality from CHD (first non-fatal event) in long-run quitters | 0.50% | 0.60% | 1.50% | 0.10% | 0.50% | 2.00% |
| Annual mortality form Stroke (first non-fatal event) in long-run quitters | 0.40% | 0.30% | 1.60% | 0.10% | 0.40% | 2.40% |
| Annual mortality from CHD (any non-fatal event) in long-run quitters | 0.70% | 0.80% | 2.50% | 0.10% | 0.50% | 2.70% |
| Annual mortality from Stroke (any non-fatal event) in long-run quitters | 0.60% | 0.40% | 2.40% | 0.20% | 0.50% | 4.10% |

*Table 4. Relative risks for disease prevalence in smokers relative to never-smokers*

|  | RR in smokers | RR in former smokers | RR in never-smokers |
| --- | --- | --- | --- |
| COPD^1^ | | | |
| M 18 - 34 | 1 | 1 | 1 |
| M 35 - 64 | 17.1 | 15.64 | 1 |
| M 65+ | 17.1^3^ | 15.64^3^ | 1 |
| F 18 – 34 | 1 | 1 | 1 |
| F 35 – 64 | 12.04 | 11.77 | 1 |
| F 65+ | 12.04^3^ | 11.77^3^ | 1 |
| Lung cancer^1^ | | | |
| M 18 - 34 | 1 | 1 | 1 |
| M 35 - 64 | 23.26 | 8.7 | 1 |
| M 65+ | 23.26^3^ | 8.7^3^ | 1 |
| F 18 – 34 | 1 | 1 | 1 |
| F 35 – 64 | 12.69 | 4.53 | 1 |
| F 65+ | 12.69^3^ | 4.53^3^ | 1 |
| CHD^2^ | | | |
| M 18 - 34 | 1 | 1 | 1 |
| M 35 - 64 | 3.35 | 1.80 | 1 |
| M 65+ | 1.60 | 1.20 | 1 |
| F 18 – 34 | 1 | 1 | 1 |
| F 35 – 64 | 4.05 | 1.85 | 1 |
| F 65+ | 1.75 | 1.20 | 1 |
| Stroke^2^ | | | |
| M 18 - 34 | 1 | 1 | 1 |
| M 35 - 64 | 3.75 | 1.1 | 1 |
| M 65+ | 1.9 | 1.1^3^ | 1 |
| F 18 – 34 | 1 | 1 | 1 |
| F 35 – 64 | 4.55 | 1.3 | 1 |
| F 65+ | 1.95 | 1.15 | 1 |

^1^Health Profile for England 2007, Department of Health, Tobacco in London, The Preventable Burden, Smokefree London & The London Health Observatory, 2004

^2^Based on CPS-II 1982-88 data which was a prospective study of smoking and death in more than 1 million Americans aged 30 and older.

^3^Relative risk assumed to be the same as that in persons aged 35-64

Formulae to calculate the expected number of cases of disease in the cohort of smokers

Total disease prevalence (
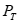
 ) within the overall population is the weighted sum of the prevalence within the three subgroups (current smokers [
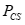
 ], former smokers [
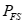
 ] and never smokers [
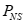
 ], with the weights being the proportion of people in each group (current smokers [
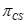
 ], former smokers [
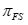
 ] or never smokers [
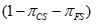
 ], Equation 1).

Equation 1


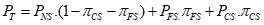


Disease prevalence within the group of current and former smokers can be expressed in terms of the prevalence among never smokers using the relative risk of the disease in current and former smokers (RR_CS_ and RR_FS_, respectively).

Equation 2


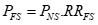


Equation 3


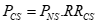


Substituting these into Equation 1 gives Equation 4, which on rearrangement, allows the disease prevalence among never smokers (
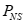
) to be expressed in terms of the total population prevalence (
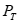
), the proportion of people who are current smokers (
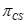
), former smokers (
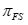
) , and the relative risk associated with being a current or former smoker (
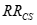
,
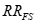
), all of which are known (Equation 5):

Equation 4


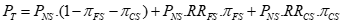


Equation 5


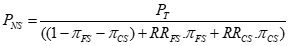


Solving for
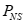
 then allows us to calculate
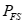
 (prevalence rate in former smokers) and
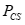
 (prevalence rate in current smokers) using Equations 2 and 3, above

Table 5. Health state costs

| **Data** | **Original source** | **n** | **Mean cost from paper (currency)** | **95% CrI (currency)** | **Cost for model (£)** | **Distribution** |
| --- | --- | --- | --- | --- | --- | --- |
| COPD | Hospital Inpatient Enquiry Database 2015. Cost per prevalent case of inpatient and day case treatment | 73,901 | 868 (Euros) | 664 to 1097  (Euros) | 1468 (Exchange rate £1 = €1.14)^124^ | Gamma (7,390,100, 0.0002) |
|  | Primary Care Reimbursement Service 2014. Cost per prevalent case of primary care treatment. | 73,657 | 662 (Euros) | 504 to 831  (Euros) |  |  |
|  | **Total of inpatient and primary care** |  | **1530 (Euros)** |  |  |  |
| Lung cancer | Hospital Inpatient Enquiry Database 2015. Cost per prevalent case of inpatient and day case treatment | 4,666 | 5107  (Euros) | 3915 to 6499  (Euros) | 5429  (Exchange rate £1 = €1.14)^124^ | Gamma (466,600, 0.01) |
|  | Primary Care Reimbursement Service 2014. Cost per prevalent case of primary care treatment | 4,666 | 555  (Euros) | 423 to 698  (Euros) |  |  |
|  | **Total of inpatient and primary care** |  | **5662 (Euros)** |  |  |  |
| CHD (non-fatal event) | British Heart Foundation. Cardiovascular Disease Statistics 2014. |  | 1323 (GBP) |  | 1460 | Gamma (100, 14.60) |
| Stroke (non-fatal event) | Xu et al, 2018 | 84,184 | 13,452 at 1 year (GBP) |  | 13,788 | Gamma (8,418,400, 0.002) |
| Asthma exacerbation | Tan et al 2016 | 939 | 341 (GBP) | SE = 12.94 | 367 | Gamma (805, 0.46) |
| Depression | Hunter et al 2013 |  | 340.35 (GBP) |  | 395 | Gamma (100, 3.95) |
| Self-harm | Tsiachristas et al 2017 | 1,140 | 809 (GBP) | SE = 26.78 | 850 | Gamma (1007, 0.84) |

*Table 6. Intervention costs*

| **Intervention** | **Assumed Mean cost (£)** | **Source** | **Assumption** |
| --- | --- | --- | --- |
| NRT Low | 83.84 | BNF | 4 weeks of 10-mg/16- hour patches and 4 weeks of 5-mg/16-hour patches |
| NRT Std | 105.65 | BNF | High-strength patch daily for 6–8 weeks, followed by medium-strength patch for 2 weeks, and low-strength patch for final 2 weeks |
| NRT High | 77.46 | BNF | 4-week supply of 21-mg Nicoderm transdermal patches, followed by 2 weeks at 14 mg and 2 weeks at 7 mg |
| Bupropion Low | 62.64 | BNF | One 150mg tablet a day for an average of 13 weeks |
| Bupropion Std | 83.52 | BNF | 150 mg daily for 6 days, then 150 mg twice daily for a period of intervention of 7–9 weeks |
| Varenicline Low | 163.8 | BNF | 0.5 mg once daily for 3 days, increased to 0.5 mg twice daily for 4 days, then 1 mg twice daily for 11 weeks |
| Varenicline Std | 163.8 | BNF | 1 mg once daily for 3 days, increased to 1 mg twice daily for 4 days, then 1 mg twice daily for 11 weeks |
| E-Cigarette | 82 | Liber et al 2017(42) | 12-week supply of e-cigarettes (e-cigarette + 3.55ml liquid per day including a replacement atomiser in months 2 and 3) |
| Bupropion Std + NRT High | 160.98 | BNF | Added cost of both interventions |
| Varenicline Low + NRT Std | 269.45 | BNF | Added cost of both interventions |
| Varenicline Std + NRT Std | 269.45 | BNF | Added cost of both interventions |
| Varenicline Std + NRT High | 241.26 | BNF | Added cost of both interventions |
| Varencline Std + Buproprion Std | 247.32 | BNF | Added cost of both interventions |

Table 7. Health state mean utility values

| **Health State** | **Utility source** | **N** | **Mean utility** | **SE** |
| --- | --- | --- | --- | --- |
| NCM, males, 18–34 years | Ara 2010 | 26,679 | 0.94 |  |
| NCM, males, 35–64 years | Ara 2010 | 26,679 | 0.88 |  |
| NCM, males, 65–100 years | Ara 2010 | 26,679 | 0.72 |  |
| NCM, females, 18–34 years | Ara 2010 | 26,679 | 0.92 |  |
| NCM, females, 35–64 years | Ara 2010 | 26,679 | 0.86 |  |
| NCM, females, 65–100 years | Ara 2010 | 26,679 | 0.70 |  |
| Lung cancer | Bertranou et al 2017 | 464 | 0.72 | 0.001 |
| COPD | Pickard et al 2007 |  | 0.69 | 0.043 |
| CHD | Stevanovic et al 206 | 30,575 | 0.76 | 0.01 |
| Stroke (first event) | Haacke et al 2006 | 77 | 0.73 | 0.036 |
| Stroke (second event) | Ara and Brazier 2010 | 18 | 0.48 | 0.087 |
| Asthma exacerbation | Lloyd et al 2007 | 112 | 0.57 | 0.026 |
| Depression | Hunter et al 2014 |  | 0.58 | 0.015 |
| Self-harm | Byford et al 2003 | 480 | 0.50 | 0.016 |

*Table 8. Expected value of perfect information and EVPPI for various subsets of model parameters, at a £20,000 willingness-to-pay value per QALY. All interventions included.*

| Model parameter subsets | EVPPI per smoker attempting to quit (£) | 1-year population EVPPI (£ million) | 5-year population EVPPI (£ million) |
| --- | --- | --- | --- |
| All (EVPI) | 3645 | 999 | 4,994 |
| All costs | 1216 | 333 | 1,667 |
| All utilities | 947 | 259 | 1,297 |
| All costs and utilities | 1415 | 388 | 1,939 |
| All abstinence probabilities | 3053 | 837 | 4,182 |
| All depression and self-harm probabilities | 1654 | 453 | 2,266 |
| E-cigarette low vs varenicline std + bupropion std (probabilities, costs and utilities) | 2342 | 642 | 3,209 |
| E-cigarette low vs varenicline std + buproprion std (probabilities only) | 1676 | 459 | 2,297 |

*Figure 1 Rank-o-grams showing the probability that each intervention is ranked 1^st^, 2^nd^, … etc. based on Net Benefit at a willingness to pay threshold of £20,000 per QALY. All interventions included.*


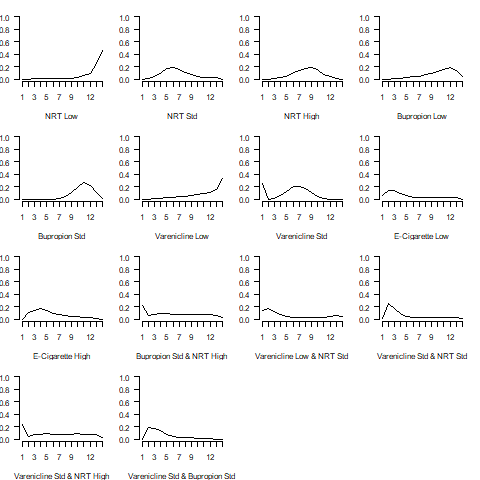

Supplement: Appendix Figure E1 and Tables E1-E8 [file mmc1.docx]
