# Supplementary material for: Cost-Effectiveness Analysis of Smoking Cessation Interventions in the United Kingdom Accounting for Major Neuropsychiatric Adverse Events
Source: Value Health. 2021 Jun;24(6):780–8. doi: 10.1016/j.jval.2020.12.012 (PMC8177405; doi:10.1016/j.jval.2020.12.012)
Supplement: Supplementary Material 2 [file mmc3.docx]

**Systematic review and network meta-analysis methods**

**Search strategy and selection criteria**

A systematic review and network meta-analysis of the effectiveness of smoking cessation medicines and e-cigarettes including only randomised controlled trials (RCTs) was conducted. NMA pools evidence from RCTs that form a network of intervention comparisons, delivering relative effect estimates that are a combination of direct (head-to-head) and indirect estimates. RCTs with duration six months or greater (≥22 weeks) in any setting in adult smokers and smokeless tobacco users were included. Studies in non-smoking populations and pregnant and breastfeeding women were excluded.

E-cigarettes and the three licensed smoking cessation medicines: varenicline, bupropion and NRT as monotherapies or in combination were included. For NRT, combinations of different formulations given concurrently (for example patch and gum) were also included. Different dosages of treatments were also examined (see Table 1). Dosage categories were determined using the British National Formulary and the MHRA public assessment report for the “e-Voke”, the first e-cigarette to be licensed as a medicine although it was later withdrawn.(1, 2)

*Table 1 Interventions by formulation and dosage*

| Treatment (formulation) | Low dose | Standard (Std) dose | High dose |
| --- | --- | --- | --- |
| Bupropion (oral extended release tablets) | <150 mg bd | 150 mg bd | >150 mg bd |
| Varenicline (tablets) | <1 mg bd | 1 mg bd | >1 mg bd |
| E-cigarette (electronic inhaler, 5 cartridges/day) | 10 mg |  | 15 mg |
| Nicotine replacement therapy (NRT) |  |  |  |
| NRT Patch (16 hours) | <15 mg | 15 mg | >15 mg |
| NRT Patch (24 hours) | <14 mg | 14 mg | >14 mg |
| NRT Gum (15/day) |  | 2 mg | 4 mg |
| NRT Nasal spray (2 sprays/hour, 64/day) |  | 0.5 mg |  |
| NRT Mouth spray (4 sprays/hour, 64/day) |  | 1 mg |  |
| NRT Lozenge (1 lozenge/1-2 hours, 15/day) | <2 mg | 2 mg | 4 mg |
| NRT Sublingual tablet (2 mg/tablet, 40/day) |  | 1/hour | 2/hour |
| NRT Inhalator |  | 10mg (12/day) | 15mg (6/day) |

The following comparators were included: placebo (reference comparator for the network meta-analysis), no drug treatment, usual care (as defined by trial authors) and wait list.

MEDLINE, Embase, PsycINFO, Web of Science, clinicaltrials.gov and Cochrane databases (Cochrane Database of Systematic Reviews, Database of Abstracts and Reviews of Effectiveness, Cochrane Central Register of Controlled Trials) and the Health Technology Assessment Database were searched with no language restrictions until February 19^th^ 2019. Reference lists of previous reviews were also manually searched and authors were contacted to identify unpublished information.

At least two reviewers screened abstracts and identified full text reports for inclusion using Covidence (covidence.org). Disagreements were resolved by reaching consensus among reviewers. Data were extracted by one reviewer on to electronic Microsoft Excel worksheets and checked by co-reviewers. Study authors were contacted in the event of missing data or unclear information. Risk of bias was assessed as high, low or unclear risk using the Cochrane risk of bias assessment tool. (3)

**Outcomes**

The primary outcome of the NMA and that used in the economic analysis was sustained (or continuous) abstinence, defined as avoidance of all tobacco use since the quit day until the time the assessment is made, occasionally allowing for lapses. Only biochemically verified (bio-verified) events were included. 171 studies (90,443 participants) reported on sustained abstinence at a follow up of at least 24 weeks, of which 161 (86,884 participants) compared two or more of the intervention classes of interest (Figure 1).

Figure 1 Network plot for sustained abstinence at class level


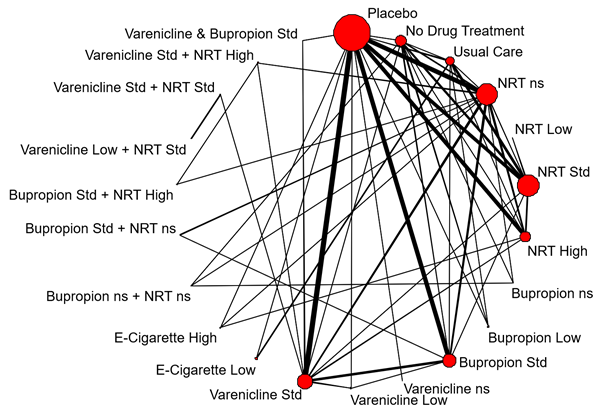


The other outcome included in the economic analysis was Major Adverse Neuropsychiatric Events (MANEs), comprising suicide, attempted suicide, suicidal ideation, depression, and seizures.^19^ Major adverse neuropsychiatric events were reported in 75 studies (42,088 patients), with 73 studies (41,483 patients) including at least one relevant comparison (Figure 2).

Figure 2 Network plot for major adverse neuropsychiatric events at class level


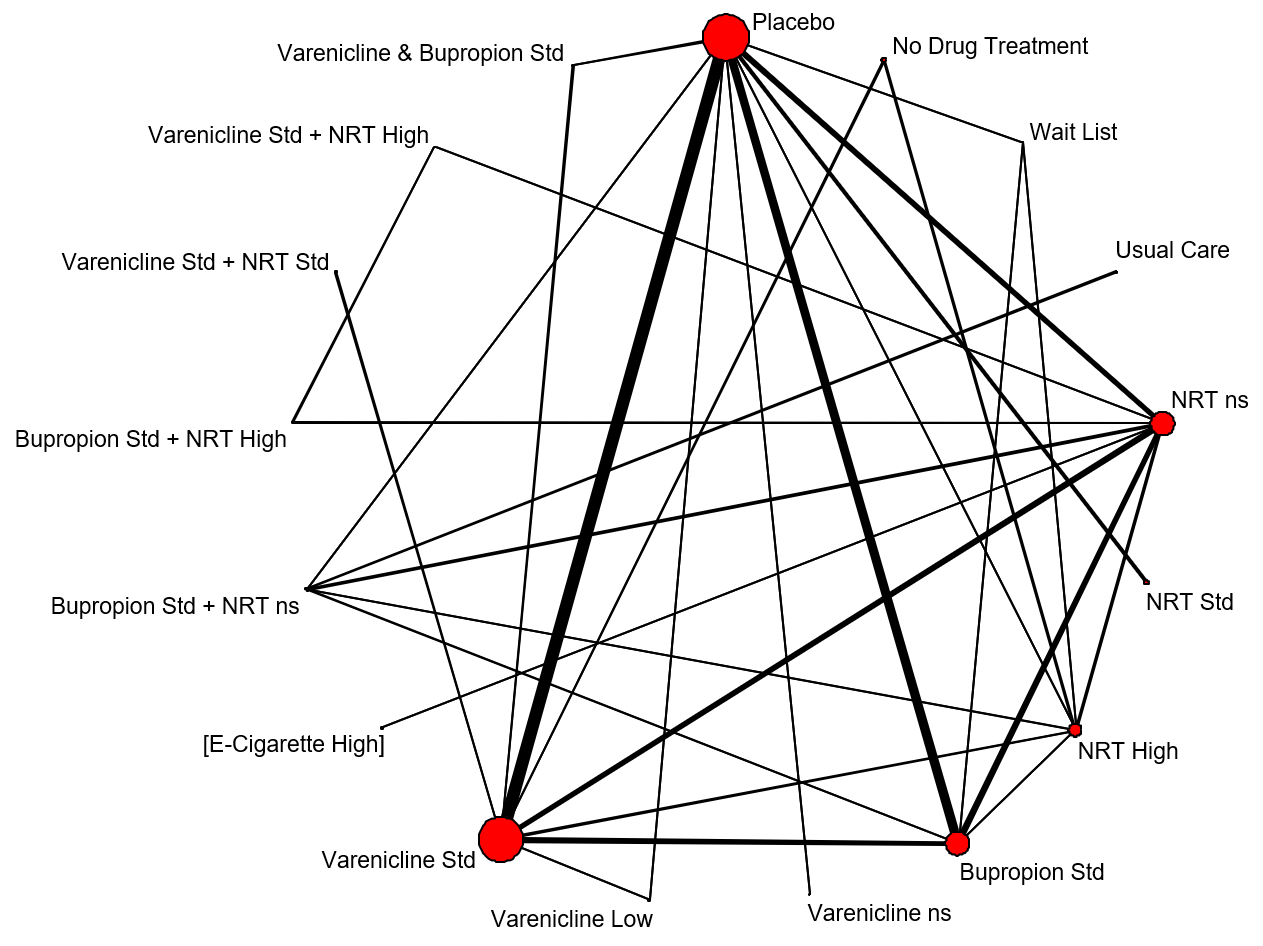


**Data analysis**

Network meta-analysis (NMA)(4) was used to combine direct and indirect evidence within a Bayesian framework using OpenBUGS (v 3.2.3). Interventions were excluded if they were disconnected from the main network. Where there were events in at least one arm of a trial but no events in one or more other arms, we added 0.5 events to all cells in the 2×2 table for that trial.(5)

NMA models for intervention classes(4) defined according to type of treatment and delivery were used (see Table 1). Due to the anticipation of heterogeneity,(7) random-effects NMAs were fitted. The tenability of the consistency assumption made in NMA was examined by comparing model fit with a model which relaxes the consistency assumption.(8) Direct and indirect estimates were also compared where both were available.

The protocol for this study is registered with PROSPERO, number CRD42016041302 and has been published.(9)

**References**

1. National Institute for Health and Clinical Excellence. British National Formulary (BNF) 2016 [Available from: <https://bnf.nice.org.uk/>.

2. Medicines & Healthcare products Regulatory Agency. Public Assessment Report. e-Voke 10mg and 15mg Electronic Inhaler (PL 42601/0003-4) 2015 [Available from: <https://mhraproductsprod.blob.core.windows.net/docs-20200330/56f25daab2a2968139bc37075e194d1a5f12b33f>.

3. Higgins JPT, Altman DG, Gøtzsche PC, Jüni P, Moher D, Oxman AD, et al. The Cochrane Collaboration’s tool for assessing risk of bias in randomised trials. 2011;343:d5928.

4. Dias S AA, Welton NJ, Jansen JP, Sutton AJ. Network meta-analysis for decision-making.: John Wiley & Sons; 2018 2018 Jan 8.

5. Sweeting MJ, Sutton AJ, Lambert PC. What to add to nothing? Use and avoidance of continuity corrections in meta-analysis of sparse data. Stat Med. 2004;23(9):1351-75.

6. Thomas KH, Dalili MN, López-López JA, Keeney E, Phillippo D, Munafò MR, et al. How do smoking cessation medicines compare with respect to their effectiveness and safety: a systematic review, network meta-analysis and cost effectiveness analysis. Health Technology Assessment. 2020.

7. Mons U, Muezzinler A, Gellert C, Schottker B, Abnet CC, Bobak M, et al. Impact of smoking and smoking cessation on cardiovascular events and mortality among older adults: meta-analysis of individual participant data from prospective cohort studies of the CHANCES consortium. BMJ. 2015;350:h1551.

8. Dias S, Welton, N.J., Sutton, A.J., Caldwell, D.M., Lu, G. & Ades, A.E. . NICE DSU Technical Support Document 4: Inconsistency in Networks of Evidence Based on Randomised Controlled Trials 2011 [updated April 2014. Available from: <http://www.nicedsu.org.uk>.

9. Thomas KH, Caldwell D, Dalili MN, Gunnell D, Munafò MR, Stevenson M, et al. How do smoking cessation medicines compare with respect to their neuropsychiatric safety? A protocol for a systematic review, network meta-analysis and cost-effectiveness analysis. 2017;7(6):e015414.
